# Modelling Hydrocortisone Pharmacokinetics on a Subcutaneous Pulsatile Infusion Replacement Strategy in Patients with Adrenocortical Insufficiency

**DOI:** 10.3390/pharmaceutics13060769

**Published:** 2021-05-21

**Authors:** Ioannis G. Violaris, Konstantinos Kalafatakis, Eder Zavala, Ioannis G. Tsoulos, Theodoros Lampros, Stafford L. Lightman, Markos G. Tsipouras, Nikolaos Giannakeas, Alexandros Tzallas, Georgina M. Russell

**Affiliations:** 1 Department of Electrical and Computer Engineering, University of Western Macedonia, 50131 Kozani, Greece; john.violaris@yahoo.com (I.G.V.); mtsipouras@uowm.gr (M.G.T.); 2 Laboratories of Integrative Neuroscience and Endocrinology, School of Clinical Sciences, University of Bristol, Bristol BS1 3NY, UK; stafford.lightman@bristol.ac.uk (S.L.L.); georgina.russell@bristol.ac.uk (G.M.R.); 3 Department of Informatics & Telecommunications, School of Informatics & Telecommunications, University of Ioannina, 47100 Arta, Greece; itsoulos@uoi.gr (I.G.T.); t.lampros@uoi.gr (T.L.); giannakeas@uoi.gr (N.G.); tzallas@uoi.gr (A.T.); 4 Centre for Systems Modelling and Quantitative Biomedicine, Institute of Metabolism and Systems Research, College of Medical and Dental Sciences, University of Birmingham, Birmingham B15 2TT, UK; e.zavala@bham.ac.uk

**Keywords:** glucocorticoid pulsatility, pharmacokinetic model, subcutaneous delivery, glucocorticoid insufficiency, hydrocortisone replacement therapy

## Abstract

In the context of glucocorticoid (GC) therapeutics, recent studies have utilised a subcutaneous hydrocortisone (HC) infusion pump programmed to deliver multiple HC pulses throughout the day, with the purpose of restoring normal circadian and ultradian GC rhythmicity. A key challenge for the advancement of novel HC replacement therapies is the calibration of infusion pumps against cortisol levels measured in blood. However, repeated blood sampling sessions are enormously labour-intensive for both examiners and examinees. These sessions also have a cost, are time consuming and are occasionally unfeasible. To address this, we developed a pharmacokinetic model approximating the values of plasma cortisol levels at any point of the day from a limited number of plasma cortisol measurements. The model was validated using the plasma cortisol profiles of 9 subjects with disrupted endogenous GC synthetic capacity. The model accurately predicted plasma cortisol levels (mean absolute percentage error of 14%) when only four plasma cortisol measurements were provided. Although our model did not predict GC dynamics when HC was administered in a way other than subcutaneously or in individuals whose endogenous capacity to produce GCs is intact, it was found to successfully be used to support clinical trials (or practice) involving subcutaneous HC delivery in patients with reduced endogenous capacity to synthesize GCs.

## 1. Introduction

Glucocorticoids (GCs) are a class of steroid hormones naturally secreted by the adrenal glands of mammals. They rapidly diffuse via the bloodstream across the body, modulating multiple physiological systems, e.g., metabolism, the immune system, the nervous system, as well as under baseline and stressful conditions [1]. Due to their lipophilic nature, GCs move freely across extracellular and intracellular membranes and bind to two classes of GC-sensitive receptors: glucocorticoid receptors (GRs) and mineralocorticoid receptors (MRs) [2]. Binding events between the hormone and its receptors induce rapid, non-genomic activity of molecular signalling cascades (involving activation and inhibition of second messenger molecules such as the cyclic adenosine monophosphate) [3] as well as delayed, genomic phenomena (activation and inhibition of gene expression after interacting with GC-responsive elements) [4]. The plethora of different cellular responses to GC stimulation is particularly evident in the brain, where both major receptor types (GRs and MRs) are exposed to GCs. In other tissues, such as the kidneys, the presence of 11β-hydroxysteroid dehydrogenase 2 in the proximity of MR- and GR-expressing cells deactivates GCs, which prevents binding to GC-sensitive receptors [5].

For any brain region containing different numbers of neurons and glial cells and expressing different distributions of GRs and MRs, the type of cellular responses to GCs can be understood as a complex interplay between: (i) the concentration of the hormone in the microenvironment of that brain region and (ii) the duration of the hormonal presence in that region. For instance, when GCs are in abundance, GRs can rapidly (i.e., within seconds) be activated by GCs and subsequently bind to chromatin. However, at each of these exchange events, when GC levels fall, there is an increased probability that the hormone ligand will be lost from the GR; unbound GR will then either be metabolized by nuclear or cytoplasmic proteosome or enter the chaperone cycle, with the association of the molecular chaperones HSP90 and p23. In this state, the GR (and other transcriptional cofactors) are not associated with chromatin, and the histone acetylation state returns rapidly to basal levels [6,7]. Moreover, the binding affinities of GCs for MRs are much higher compared to GRs—i.e., lower levels of GCs are required to induce MR-dependent effects, whereas GR-dependent effects require higher GC levels [8]. Finally, prolonged exposure to high GC levels leads to downregulation of GR expression in the brain [9]. These examples indicate how important the dynamic regulation of GC levels in the microenvironment of brain cells is for determining the type of cellular responses to GC stimulation.

The delayed negative feedback loop between the control centre of GC biosynthesis (anterior pituitary) and the GC-producing tissue (adrenal gland cortex) creates a natural, ultradian rhythm of GC secretion [10]. This ultradian rhythm consists of periodic bursts of GC biosynthesis and then releases into the systemic circulation, which rapidly travels across the body (95% of which is bound to carrier proteins of the blood stream). Thus, under baseline conditions, GC-sensitive tissues (such as the brain) are exposed to short-lasting GC pulses, wherein periods of high GC levels (potentially inducing genomic and non-genomic GR-dependent and non-genomic MR-dependent effects) alternate with periods of low GC levels (crucial for resetting the afore-mentioned molecular mechanisms, i.e., sustain their sensitivity to a forthcoming GC pulse) [11]. The ultradian GC rhythm is not only significant from a physiological point of view but also from a pharmacological perspective: current GC replacement therapy does not reproduce the characteristics of complex physiological GC rhythms, leading to suboptimal treatment of patients with adrenocortical insufficiency [12].

In recent years, we conducted two clinical trials to investigate the neurobiological significance of the ultradian GC rhythm in humans, either healthy volunteers [13,14] or patients with adrenocortical insufficiency. The trial in the latter case [15] aimed at altering the HC dosing regimen to restore physiological ultradian hormonal rhythmicity. These studies were the first to show that something closer to physiological HC delivery might improve brain function in patients and offer a better self-perceived sense of well-being and quality of life. In both studies, we used a novel pump of sub-cutaneous HC delivery (Crono P^®^, CANE Applied Medical Technology Ltd., Cambridge, UK), programmed to deliver 8 HC pulses every three hours at a rate of 10 μL/s [14], and in particular 400 μL solution (containing 4 mg of hydrocortisone) at 03:00 a.m., 06:00 a.m. and 09:00 a.m., 230 μL solution (containing 2.3 mg of hydrocortisone) at 12:00 p.m., 15:00 p.m., and 18:00 p.m., and 50 μL solution (containing 0.5 mg of hydrocortisone) at 21:00 p.m. and midnight. A preliminary validation study preceded these trials to verify that pharmacological interventions involving the Crono P^®^-mediated HC delivery could achieve the desired GC dynamics in the blood stream [16].

The dynamics of GCs in the systemic circulation created by the Crono P^®^ under different settings has been previously monitored through daily blood sampling sessions with a 10 min sampling rate, as well as a subsequent determination of plasma cortisol levels. However, this process is enormously labour-intensive for both examiners and examinees and its feasibility is questionable under certain experimental settings (e.g., multimodal neuroimaging studies that can take up to two hours, including preparation of the participants, potential cognitive training, scanning and/or neurofeedback session). Thus, the aim of this work is to provide a mathematical model that describes the pharmacokinetics of the subcutaneous delivery of HC doses through the Crono P^®^. We show how this model can be used to approximate the values of plasma cortisol levels at any time point of the day using only a limited number of plasma cortisol measurements as model inputs.

## 2. Results

### 2.1. HC Delivery Assuming Constant Absorption and Clearance Rates

**Figure 1:**
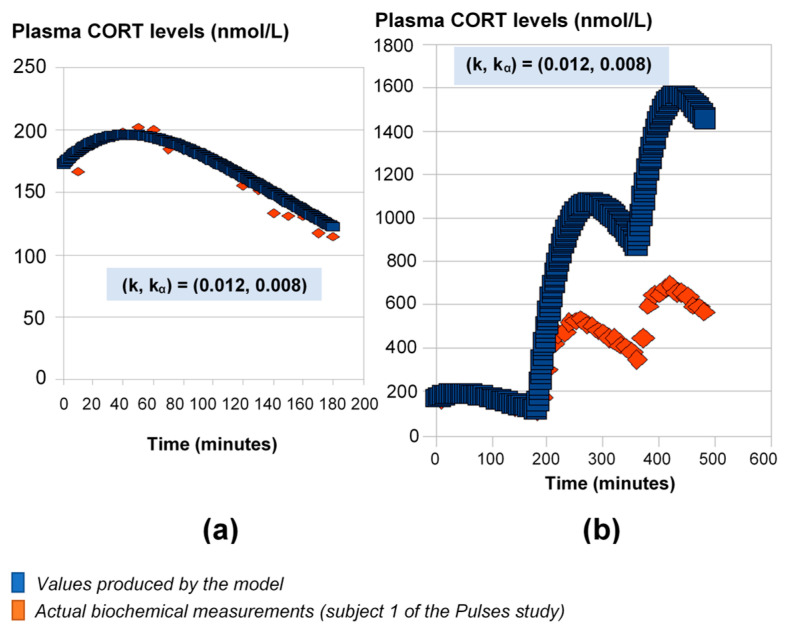
Mathematical model predictions of plasma cortisol levels assuming constant absorption (kα) and secretion (k) rates. The model fits the data during the first three hours (**a**), but for the estimated (k, k_α_) values, it significantly deviates from the observed plasma cortisol values past the first three hours (**b**). Plasma cortisol measurements belong to Subject 1 from the Pulses study (see Materials and Methods). CORT: cortisol.

For this part of the study, we used the 24-h plasma cortisol profile of Subject 1 from the Pulses study (see Materials and Methods). First, we fitted the model parameters k and k_α_ using the initial 3 h of data (Figure 1a). If we continue the model simulations for the next 6 h of data using these rates, the model predictions diverge from the measured levels of plasma cortisol (Figure 1b).

### 2.2. HC Delivery Assuming Piecewise Absorption and Clearance Rates That Change Every 3 h

**Figure 2:**
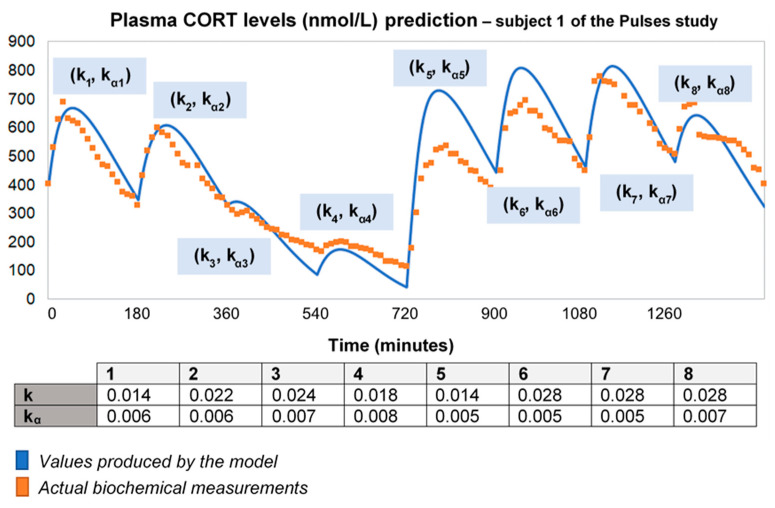
To improve the fitting of the model for longer time intervals, we recalibrated the absorption and clearance rates every 3 h. Specifically, we used the model to produce a curve for every 3-h interval, which was then fitted to data with different (k, k_α_) pairs for each curve. Then, we concatenated the curves creating a piecewise fitted simulation with a duration of 24 h. Time points of delivery (magnitude of doses): 0 min (2.3 mg/230 μL), 180 min (2.3 mg/230 μL), 360 min (0.5 mg/50 μL), 540 min (0.5 mg/50 μL), 720 min (4 mg/400 μL), 900 min (4 mg/400 μL), 1080 min (4 mg/400 μL) and 1260 min (2.3 mg/230 μL). As before, plasma cortisol measurements from Subject 1 of the Pulses study were used. CORT: cortisol; k: clearance rate; k_α_: absorption rate.

To improve the output of the model for longer time intervals (using the 24-h plasma cortisol data of Subject 1 from the Pulses study), we needed to recalibrate the absorption and clearance rates every 3 h. This piecewise adjustment of model parameters improves the model fitting to data over 24 h (Figure 2). However, this is not an adequate process for a predictive model with minimal input.

### 2.3. HC Delivery under Discretely Varying Absorption and Clearance Rates (Optimised Model)

**Figure 3:**
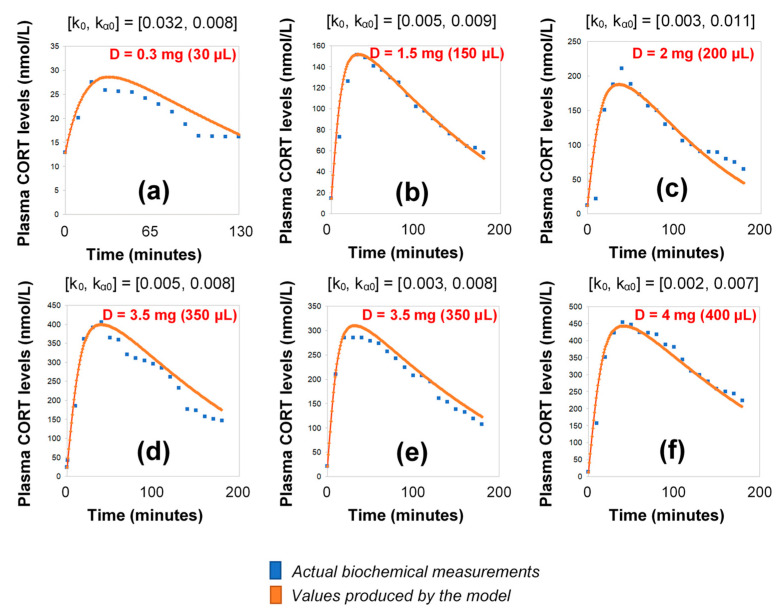
Simulation of plasma cortisol changes in different healthy subjects with pharmacologically suppressed cortisol biosynthesis with dexamethasone (see Materials and Methods) after the subcutaneous infusion of varying doses of hydrocortisone. The simulations were produced by the revised model (see Materials and Methods), which assumes discretely varying absorption and secretion rates of cortisol. (**a**) female subject, BMI: 20.4, pulse time: 10:00 a.m., pulse volume (D): 30 μL (0.3 mg); (**b**) male subject, BMI: 30, pulse time: 10:00 a.m., pulse volume (D): 150 μL (1.5 mg); (**c**) male subject, BMI: 22.5, pulse time: 10:30 a.m., pulse volume (D): 200 μL (2 mg); (**d**) male subject, BMI: 21.2, pulse time: 09:50 a.m., pulse volume (D): 350 μL (3.5 mg), (**e**) male subject, BMI: 23.3, pulse time: 10:10 a.m., pulse volume (D): 350 μL (3.5 mg); (**f**) female subject, BMI: 23.4, pulse time: 09:50 a.m., pulse volume (D): 400 μL (4 mg); BMI: body-mass index, CORT: cortisol, D: dose.

**Figure 4:**
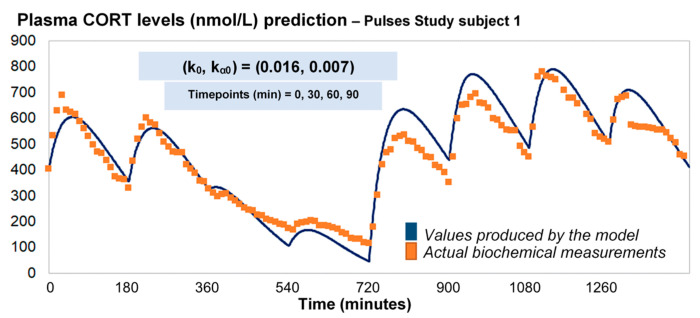
Assuming discretely varying absorption and clearance rates (revised model), the brute-force search method provides the initial pair of (k_0_, k_α0_), based on four CORT measurements (e.g., at time points 0, 30, 60 and 90 min), and subsequently estimates the whole 24-h profile of plasma cortisol. The data from the Pulses study Subject 1 was used (see Materials and Methods). CORT: cortisol, k: clearance rate, k_α_: absorption rate. Time points of delivery (magnitude of doses): 0 min (2.3 mg/230 μL), 180 min (2.3 mg/230 μL), 360 min (0.5 mg/50 μL), 540 min (0.5 mg/50 μL), 720 min (4 mg/400 μL), 900 min (4 mg/400 μL), 1080 min (4 mg/400 μL), 1260 min (2.3 mg/230 μL).

Under the assumption of discretely varying absorption and clearance rates, we proceeded to recalibrate the model by computing just a single pair of initial (k_0_, k_α0_) for each individual (rather than every 3 h). To do this, we first used six 3-h healthy volunteer profiles with endogenous cortisol secretion suppressed by dexamethasone (Figure 3). Subsequently, we replicated the 24-h plasma cortisol profile of Subject 1 from the Pulses study by just estimating a single, initial pair of (k_0_, k_α0_). Specifically, we estimated k_0_ = 0.016 and k_α0_ = 0.0074 from only 4 plasma cortisol measurements (t_0_, t_0_ + 30 min, t_0_ + 60 min and t_0_ + 90 min). We chose t_0_ as the first cortisol measurement (which was concurrent with the 15 h pulse from the pump) (Figure 4).

As a next step, we wanted to see how our model would behave if we chose a different combination of four cortisol measurements. We performed 7 runs, retaining the temporal relationship between the measurements (t_0_, t_0_ + 30 min, t_0_ + 60 min and t_0_ + 90 min) but shifting t_0_ by 3 h every time to concur with a different pulse of the pump (t_0_ = 180, 360, 540, 720, 900, 1080, 1260) (Appendix A). Finally, we performed 3 additional runs, retaining t_0_ = 0 but changing the time distance between the four cortisol measurements: (1) reducing their in-between distance (t_0_, t_0_ + 20 min, t_0_ + 40 min and t_0_ + 60 min), (2) increasing their in-between distance (t_0_, t_0_ + 120 min, t_0_ + 720 min and t_0_ + 1090 min) and (3) having two distant pairs of cortisol values, each pair containing two proximal values (t_0_, t_0_ + 50 min, t_0_ + 300 min and t_0_ + 350 min) (Appendix B).

### 2.4. Validation of the Optimised Model for HC Delivery

**Figure 5:**
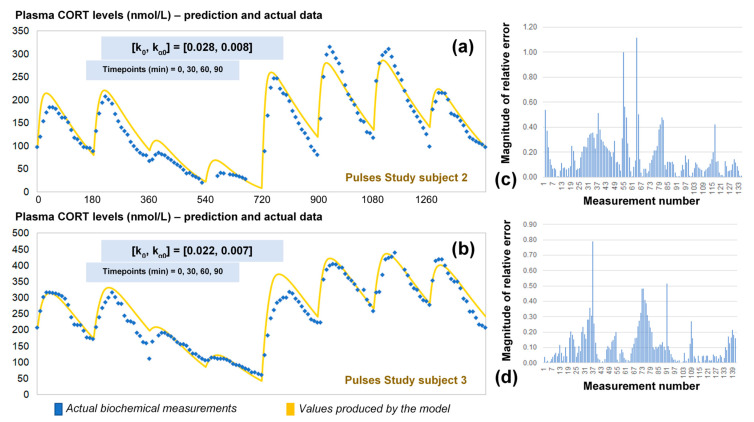
(**a**) Comparison of the revised model predictions (yellow line) with the ultradian rhythm of plasma cortisol observed in subject 2 (blue dots) from the Pulses study following subcutaneous pulsatile CORT infusion (see Materials and Methods). (**b**) Comparison of the revised model predictions (yellow line) with the ultradian rhythm of plasma cortisol observed in subjects 3 (blue dots) from the Pulses study following subcutaneous pulsatile CORT infusion (see Materials and Methods). The revised model assumes discretely varying absorption and clearance rates and the brut-force search method calculates the initial pair of (k_0_, k_α0_) rates by four cortisol measurements (for instance at timepoints 0, 30 60 and 90 min). We also estimated the relative error of the model predictions across all plasma cortisol data points for subject 2 (**c**) and 3 (**d**). The periodicity of the data allows us to assume that the value of cortisol 24 h later is the same as the first value given, and hence, the error is reduced as we approach the end of that 24-h period. CORT: cortisol, k: clearance rate, k_α_: absorption rate. Time points of delivery (magnitude of doses): 0 min (2.3 mg/230 μL), 180 min (2.3 mg/230 μL), 360 min (0.5 mg/50 μL), 540 min (0.5 mg/50 μL), 720 min (4 mg/400 μL), 900 min (4 mg/400 μL), 1080 min (4 mg/400 μL), 1260 min (2.3 mg/230 μL).

The revised assumptions enabled the model to estimate plasma cortisol data through a simple and easy to use algorithm requiring only four data points as inputs. To validate our approach, we predicted the 24-h cortisol profile from the remaining two participants from the Pulses study (Figure 5). We did this by calculating the corresponding single pair of (k_0_, k_α0_) rates from only four plasma cortisol values measured at time points 0, 30 min, 60 min and 90 min. We also estimated the magnitude of the relative error for every time point of the model predictions versus the plasma cortisol data. Finally, the mean absolute error was estimated to be 14%.

### 2.5. Reaches and Limitations of the Model

A question that naturally arises is what is the best possible fit that our model can achieve. The process of finding this is straightforward given the following functions:

(1) $$C_{n}\left(t,k_{0},k_{a0}\right)=\frac{k_{a}\left(t\right)h}{k_{a}\left(t\right)-k\left(t\right)}\left(\overset{n}{\underset{i=0}{\sum }}\delta_{i}\left(t\right)D_{i}\left(e^{-k\left(t\right)\left(t-t_{i}\right)}-e^{-k_{a}\left(t\right)\left(t-t_{i}\right)}\right)\right)+\sigma e^{-k\left(t\right)t}$$

*M_i_(t)* is the *i*-th measurement taken every 10 intervals. We defined the error function for any *n* measurements as follows:

(2) $$E_{f}\left(k_{0},k_{a0}\right)=\overset{n}{\underset{i=0}{\sum }}\frac{1}{n}{\left(C_{i}\left(t,k_{0},k_{a0}\right)-M_{i}\left(t\right)\right)}^{2}$$

Therefore, we are interested in finding the ordered pair (*k*_0_, *k*_*α*0_) that minimizes this function, something that is achievable through basic multivariable calculus techniques.

## 3. Discussion

The use of smart, automated pumps for subcutaneous drug delivery is likely to expand over the next few decades as chronopharmacological research continues to provide new evidence on their therapeutic efficacy [17]. In various pathological conditions, smart pumps can improve drug administration by splitting it into multiple parts throughout the day (even during sleep), and by adjusting dose magnitude according to the time of the day and the patient’s internal time (e.g., pre-prandial vs. post-prandial states). These concepts are especially relevant to hormonal replacement therapies.

For the last 15 years, the scientific community has gathered multiple complementary pieces of evidence that support the notion that glucocorticoid pulsatility has biological significance [18], and thus chronopharmacological aspects in therapeutics involving glucocorticoids should be taken into serious consideration. In this context, pump-mediated glucocorticoid replacement strategies can replicate fast hormone secretion dynamics, thus offering certain advantages over other treatment approaches that disregard ultradian hormone pulsatility (e.g., oral substitution of HC twice or thrice daily or a pump-mediated continuous HC replacement).

Our group examined the scientific potential and challenges of pump-mediated dynamic hormone infusion through a series of clinical trials [13,16,19,20]. In these trials, either healthy volunteers with a pharmacologically disrupted capacity to produce glucocorticoids or patients with adrenocortical insufficiency underwent a pulsatile scheme of subcutaneous HC replacement. In the case of patients, this involved the daily infusion of 8 pulses of HC every 3 h, with varying doses depending on the time of the day. In these studies, patients were required to remain hospitalized for at least 24 h, while repeated blood samples were collected throughout the day. This continuous sampling allowed us to construct their daily plasma cortisol profile, and thus verify whether the pump-mediated treatment was replicating the desired hormonal dynamic pattern.

In this paper, we postulated a mathematical model that describes the pharmacokinetics of cortisol administration in adrenocortical insufficiency patients. We show how the model can be used to calibrate pump-mediated HC replacement, a necessary step for its adoption in clinical practice. Further, we presented an automated process to recalibrate the pump that optimizes plasma cortisol delivery for any individual given only four single time point measurements of plasma cortisol. In general, our model shows a reasonable accuracy (mean absolute percent error of 14%) in predicting 24 h long hormone profiles measured in patients (Figure 5). For smaller periods of time, the model accuracy can improve notoriously as seen from the simulations that lasted for 3 h (Figure 3). It is expected that small errors in our parameter estimations would invalidate the model predictions for long periods of time. Yet our model can give a good estimate for the pharmacokinetics of cortisol replacement in pump-mediated clinical trials that last up to 24 h.

Mathematical modelling can also shed light on our understanding of the physiological mechanisms underlying the dynamic absorption and clearance of subcutaneous HC. While both rates are likely to depend on individual differences (such as body mass index), our model suggests that the clearance rate varies throughout the day. During our model recalibration, we found the clearance can be affected by baseline levels of cortisol already in the bloodstream, while at the same time the rate of absorption seems to drop the more HC it is administered. The estimated rate of cortisol clearance is well within the predicted variability observed in previous studies [21]. It should also be noted that, while k can vary wildly among individuals, our model suggests that k_α_ variability arises less from individual differences and depends more on the given HC dose. On the other hand, the optimised model relies on the assumption that although the absorption and clearance rates change over time, these changes are discontinuous and therefore the constant rate model is a good approximation for small intervals of time. The algorithm used to estimate the initial (k, k_α_) rates performs better when the input data points lie within a 30–40-min range, when they are associated to low cortisol levels and when at least one local maxima of the plasma cortisol profile is included. Similarly, the algorithm assumes that after 24 h the cortisol levels must return to a value close to the initial data point. This considerably minimises the error for the last 3 h of simulation. In general, the highest error is observed the further the distance from the initial data points and before the last 6 h of the 24-h period (i.e., at the interval from the 6-th to the 18-th hour from the beginning of the simulation).

Lastly, it is important to highlight that our model cannot predict plasma cortisol pulsatility if HC is administered in ways other than subcutaneously, or in individuals whose endogenous capacity to produce glucocorticoids is intact. This is because the well-defined subcutaneous infusion scheme would be confounded–both dynamically and quantitatively, with endogenous bursts of glucocorticoid secretion (affected by individual differences in lifestyle, gene expression, and experience of stressful events). The model may be solely used to predict and calibrate the pharmacokinetics of subcutaneous HC infusions in individuals with impaired endogenous steroidogenic capacity.

## 4. Materials and Methods

### 4.1. Participants and Interventions

We extracted plasma cortisol data from clinical trials performed at the University of Bristol (UK) over the past 7 years. These trials were performed in accordance with the highest bioethical and local institutional provisions, under the approval of the Medicines and Healthcare Products Regulatory Authority and according to the principles of Good Clinical Practice and the Declaration of Helsinki. These studies recruited: (1) patients with adrenocortical insufficiency (Addison’s disease and congenital adrenal hyperplasia) (Pulses study, IRAS ID: 98045, EudraCT No: 2012-001104-37, ISRCTN67193733), and (2) healthy volunteers whose endogenous cortisol secretion was suppressed via dexamethasone administration (IRAS ID: 106181, UKCRN-ID-15236). In the latter case, 1 mg of dexamethasone was received orally by healthy volunteers at midnight at their home, and 1 mg was administered at 9 a.m. the next day at the research unit, prior to the experiment. During the experiment, volunteers received two subcutaneous infusions of HC (via CANE pump, see below) at varying time intervals and doses (dose range 0.3–4 mg) over a 7-h period [16]. The first 3 h of plasma cortisol data following the first HC pulse were used in this study. In the case of patients with adrenocortical insufficiency, HC replacement therapy was administered over the course of 24 h in the same way (subcutaneously via the CANE pump, see below), but distributed in 8 pulses (i.e., doses) with a constant 3 h inter-pulse interval and three different dose amplitudes, a high dose (4 mg) at 03:00 a.m., 06:00 a.m. and 09:00 a.m., an intermediate dose (2.3 mg) at midday, 03:00 p.m. and 06:00 p.m. and a small dose (0.5 mg) at 09:00 p.m. and midnight [13].

### 4.2. Technical Specifications of Subcutaneous Hydrocortisone Delivery

A portable subcutaneous infusion pump (Crono P, CANE Applied Medical Technology Ltd., Cambridge, UK) containing 100 mg of HC in 1 mL (Efcortesol^®^; Sovereign Medical Ltd., Stansted, Essex, UK) was made up to 10 mL with 0.9% saline and programmed to deliver a high-, medium- and low-sized pulse of HC. All pulses were delivered at a flow rate of 10 μL/s. The pump delivered HC subcutaneously via a cannula (Medtronic quick-set^®^, Medtronic MiniMed, Northridge, CA, USA) inserted into the abdominal subcutaneous tissue.

### 4.3. Acquisition of Plasma Cortisol Data

During the experimental process, blood samples were collected via an intravenous cannula every 10 min for serum cortisol concentration estimation. Blood sampling was being performed by the human automated blood sampler (HABS) [19]. The latter is a multi-device system, controlled by a computer, dedicated to collect blood samples and substitute the amount of blood received with an equal amount of normal saline (containing NaCl 0.90% *w*/*v*). After each sampling session, samples were allowed to clot at room temperature prior to centrifugation and serum was frozen at −80 °C until assayed. Analysis was performed by an electrochemiluminescence immunoassay (Cobas^®^, Roche Diagnostics Ltd., West Sussex, UK). Cross reactivity with 11-deoxycortisol was 4.1%.

### 4.4. Model Validation Strategy

**Figure 6:**
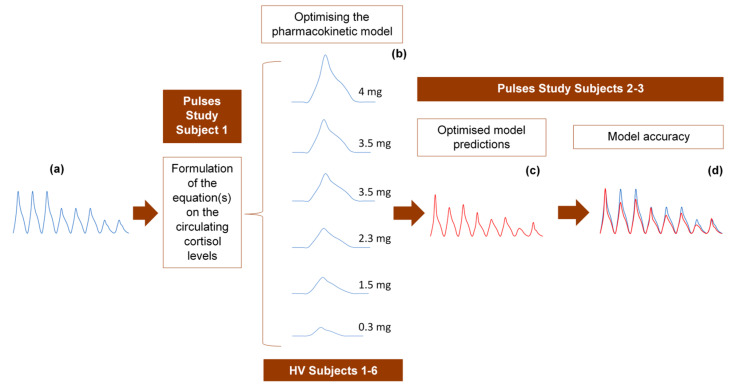
Strategy for developing and optimising the pharmacokinetic model, describing the subcutaneous delivery of hydrocortisone (HC). (**a**) One of the 24-h cortisol profiles (blue diagram) was selected at random and used to formulate an equation describing plasma cortisol dynamics. We assumed constant rates of HC absorption and clearance and showed that such an approach cannot simulate reality. Three different scenarios were subsequently tested, of which only one was finally selected. (**b**) The selected optimised scenario assumes varying rates of HC absorption and clearance depending on the dose magnitude and circulating cortisol levels, respectively. The six 3-h healthy volunteer profiles (blue diagrams) under different subcutaneous doses of HC were used to validate these assumptions and confirm the reliability of the model. (**c**) Four plasma cortisol values from each of the remaining two patients with adrenocortical insufficiency were inputted into the model to predict their whole 24-h hormonal profile (red diagram). (**d**) The (theoretical) output of the model (red diagram) was compared to the actual 24-h plasma cortisol profile (blue diagram) of each of the two patients. HV: healthy volunteers.

The 24-h hormone profiles of 9 participants (6 healthy volunteers and 3 adrenal insufficiency patients) were used to validate the model as follows: randomly, one of the 24-h profiles was initially used to formulate the equation describing the circulating plasma cortisol concentration as a function of time. The 6 profiles from the healthy volunteer study were subsequently used to refine the pharmacokinetic model through model parameter estimations, optimisations and validation of the assumptions related to the absorption and clearance of cortisol. Finally, we tested the accuracy of the model to replicate the data from the two remaining 24-h profiles of the Pulses study (Figure 6).

### 4.5. Description of Parameters

Table 1 describes all parameters and state variables used in this work.

### 4.6. Mathematical Modelling

**Figure 7:**
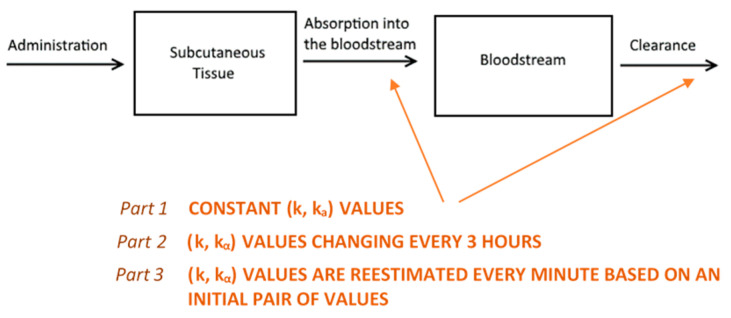
An illustration of the reasoning behind the two-compartment model. Hydrocortisone (HC) is administered into the subcutaneous tissue where it is absorbed with a rate of *k_α_*. Then, it is distributed via the bloodstream throughout the body, where it is cleared (by the liver and kidneys) with a rate *k* as predicted by the model. Initially, we assumed that both absorption and clearance rates were constant (part 1) and demonstrated why this is an unrealistic assumption. Following this, we proceeded to estimate a different ordered pair (*k*, *k_α_*) for every three-hour interval. Then, we concatenated the eight curves together to create a graph for a full day (part 2). Finally, we assumed that both rates change every minute (part 3), with the absorption rate depending on the magnitude of the dose administered and clearance rate depending on circulating cortisol levels.

We based the formulation of the pharmacokinetic model on established biochemical principles [22]. Our approach involves a two-compartment model of sub-cutaneous and plasma GC dynamics. Starting from the simple assumption that GC absorption and clearance rates are constant (Part 1, see below), we proceed to show how considering them as variable improves the model predictive power (Parts 2 and 3, see below) (Figure 7). In all model simulations, we assumed a constant plasma volume of 3.025 L [23]. All simulations were produced using a computer program written in the C++ programming language.

#### 4.6.1. Part 1

Initially, we assumed that both the rates of absorption and clearance are constant, and postulated the following system of ordinary differential equations to describe the underlying dynamics:

(3) $$\frac{dS_{c}\left(t\right)}{dt}=-k_{a}S_{c}\left(t\right)$$

(4) $$\frac{dC\left(t\right)}{dt}=-kC\left(t\right)+k_{a}S_{c}\left(t\right)$$

where *S_c_(t)* and *C(t)* are the cortisol concentration in subcutaneous tissue and plasma, respectively. Both are measured in nmol/L. The rates of absorption and clearance are symbolized with *k_α_* and *k*, respectively, and are considered constant.

Equation (3) is easily solvable, with

(5) $$S_{c}\left(t\right)=De^{-k_{a}t}$$

where *D* stands for the delivered HC dose measured in mg.

Substituting Equation (5) in (4) leads to the single differential equation:

(6) $$\frac{dC\left(t\right)}{dt}+kC\left(t\right)=k_{a}De^{-k_{a}t}$$

The solution to Equation (6) is derived in Appendix C and has the form:

(7) $$C\left(t\right)=D\frac{k_{a}}{k_{a}-k}\left(e^{-kt}-e^{-k_{a}t}\right)+\sigma e^{-kt}$$

where *σ* denotes the initial cortisol measurement in plasma (*σ* = *C*(0)).

To predict the concentration of cortisol for an arbitrary number of sequential dosages, we generalize Equation (7) to:

(8) $$C_{n}\left(t\right)=\frac{k_{a}h}{k_{a}-k}\left(\overset{n}{\underset{i=1}{\sum }}\delta_{i}\left(t\right)D_{i}\left(e^{-k\left(t-t_{i}\right)}-e^{-k_{a}\left(t-t_{i}\right)}\right)\right)+\sigma e^{-kt}$$

where *n* is the number of dosages and *h* is a factor that converts the units of cortisol concentration from mg to nmol/L. The function *δ_i_(t)* is a step function selecting for the *i*-th dose, with *δ_i_(t)* = 1 if *t* ≥ *t_i_* and *δ_i_(t)* = 0 if *t* < *t_i_*. The derivation of Equation (8) can be found in Appendix D. Using Equation (8), we simulated HC replacement throughout an entire day while keeping (*k*, *k_α_*) constant.

#### 4.6.2. Part 2

Using Equation (8), we produced a solution curve for every 3-h interval, which was fitted to the data by choosing different (*k*, *k_α_*) pairs for each curve. Then, we concatenated the curves creating a fitted simulation with a duration of 24 h.

#### 4.6.3. Part 3

**Figure 8:**
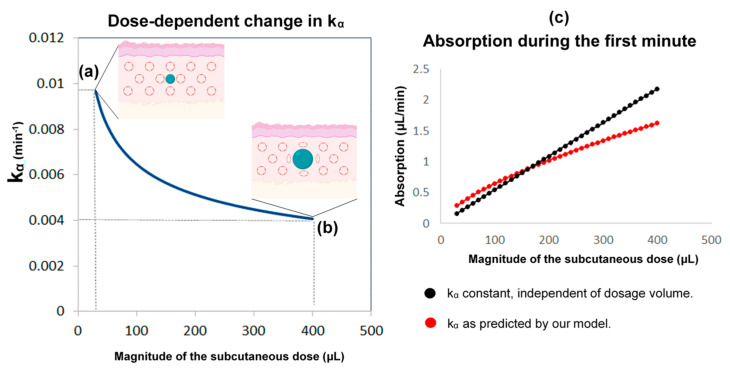
The reasoning behind the assumptions regarding the absorption rate of subcutaneous hydrocortisone (HC) administration. This rate depends on the subcutaneous dose magnitude, and the within-subject change in absorption rate with time. HC is a lipophilic molecule; its diffusion is hardly restricted by tissue membranes and thus can be assumed to be isotropic. Accordingly, we used a sphere as an approximation of the shape and movement of hormonal molecules infused into the subcutaneous tissue. **Left**: the higher the subcutaneous dose volume, the lower the absorption rate k_α_ (k_α_ is inversely proportional to the cubic root of the dose volume). When a small dose is administered by the pump cannula (e.g., 30 μL or 0.3 mg of HC) (**a**), the ratio of the area of the sphere to its volume is approximately 2.4-times greater than when a 13-times larger dose is administered (e.g., 400 μL or 4 mg of HC) (**b**). Therefore, the absorption rate drops to almost a similar degree (from about 0.01 to about 0.004). When administered in high doses, an increased fat deposition of the hormone could also contribute to dose-dependent changes in the absorption rate. **Right**: compared to the state where the absorption rate was considered constant and independent of the dosage, our assumption (of a dose-dependent k_α_) indicates that in lower doses, the absorption would be higher, while in higher doses would be lower (**c**). Part of the Figure has been created with BioRender.com.

In this part, we developed a more realistic model by introducing the following considerations: (i) Although the absorption and clearance rates change over time, these changes are discontinuous and hence the constant rate model can be a good approximation for small intervals of time. (ii) The clearance rate *k* varies proportionally to the concentration of cortisol in plasma. Furthermore, the assumption that better approximates the data is to use the maximum value of *k(t)* of all the values that have been calculated until now as shown below; which resets to its initial value *k*_0_ every 24 h at 12:00 noon (in other words, *k(t)* can only increase, but not decrease, proportionally to *C(t)* until it resets every 24 h). (iii) The rate of absorption (*k_α_*) depends on the moving boundary of a small sphere of liquid created within the subcutaneous tissue following HC delivery (Figure 8).

Based on consideration (ii), it follows that for *K(t)*, a function such that $K\left(t\right)=k_{0}\frac{C\left(t-1\right)}{C\left(0\right)}$ for some initial concentration *C*(0) and for x∈R, the plasma cortisol clearance rate is given by k(t)=max{K(x):0<x≤t−1} if *t >* 0 and *k(t)* = *k*_0_ if *t* = 0. Then, if *t* = 1260 min (at 12:00 p.m.), k(t) = $k_{0}$ (see also Appendix E). In relation to consideration (iii), we hypothesized that a small spherical pressure region is created within the subcutaneous tissue following the infusion of the HC solution [24,25,26,27].

The rate of absorption is dependent on the area of that receding sphere. Since the absorption rate is calculated as a percentage of cortisol at a given time, we postulated the following formula for time t and some constant $\hat{v}$:

(9) $$k_{\alpha }\left(t\right)=\frac{4\pi r{\left(t\right)}^{2}}{V}\hat{v}$$

The radius of the sphere in time *t* is denoted by *r*(*t*):

(10) $$k_{a}\left(t\right)=\frac{4\pi \hat{v}{\left(r\left(t\right)\right)}^{2}}{\frac{4}{3}\pi {\left(r\left(t\right)\right)}^{3}}=\frac{3\hat{v}}{r\left(t\right)}$$

Since each HC dose is delivered by the infusion pump in the same place, *r(t)* is dependent on the dosage *S_c_(t)* that remains in the subcutaneous compartment, which is a function of time. Taking into consideration that 1 mg of HC is contained in 100 μL of solution, *r(t)* can be expressed as:

(11) $$r\left(t\right)={\left(\frac{75}{\pi }\times S_{c}\left(t\right)\right)}^{\frac{1}{3}}$$

It follows that $k_{a}\left(t\right)={\left(\frac{S_{c}\left(0\right)}{S_{c}\left(t\right)}\right)}^{\frac{1}{3}}\times k_{a}\left(0\right)$ for some initial *k_α_*(0) and initial dosage *D*(0) (see Appendix F).

Finally, based on our initial assumptions about *k_α_* being dependent on the moving boundary of a small sphere, we estimated the volume in the subcutaneous tissue (see Appendix G) for any given time. To make calculations easier, we did that by estimating it once every minute.

A brute-force search algorithm checks the output of the model for different initial pairs of (*k*_0_*, k_α_*_0_), to minimize the error with the four actual cortisol values given as input, and then the whole 24-h curve is produced based on the chosen pair of (*k*_0_, *k_α_*_0_). Following this, the pair of (*k, k_α_*) for every subsequent minute is calculated based on the algorithms provided in Appendix E and Appendix G.

## 5. Conclusions

In this paper, we introduce a pharmacokinetic model that predicts the 24-h plasma cortisol profile for patients with adrenocortical insufficiency under subcutaneous pulsatile HC replacement with good accuracy (mean absolute percent error of 14%), especially when only four plasma cortisol measurements are provided. This pharmacokinetic model can be used to support clinical trials or practice involving subcutaneous HC delivery in patients with reduced endogenous capacity to synthesize GCs.

## Figures and Tables

**Table 1:** Biomodelling Parameters.

Parameter	Description	Means of Estimation	Units
C(t)	Plasma cortisol concentration as a function of time.	Predicted by the model	nmol/L
S_c_(t)	Subcutaneous cortisol concentration as a function of time.	Predicted by the model	mg
D_i_	Hydrocortisone dosage	Known from the experiment	mg
k_α_(t)	Cortisol absorption rate in subcutaneous tissue. Real positive number.	Initial value fitted to data	1/min
k(t)	Plasma cortisol clearance rate. Real positive number.	Initial value fitted to data	1/min
t	Time	Self-explanatory	min
σ	Initial cortisol levels in the subjects’ bloodstream (C(0))	Known from the experiment	nmol/L
h	Unit conversion factor for concentration from mg to nmol/L in plasma. Real number, constant, h = 10^6^/(362.42 × ℓ)	Self-explanatory	nmol/mg
ℓ	Plasma volume of a typical subject.	Estimated from the literature.	L

**Figure A1:**
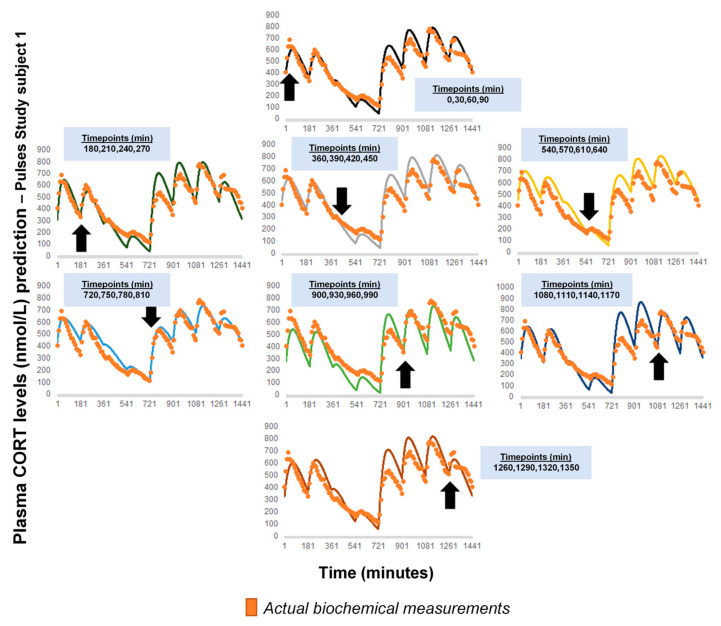
Comparison of the revised model predictions with plasma CORT data, if calculation of the initial pair of (k, kα) was based on another set of four CORT time point measurements. The time distance between the four CORT measurements was retained (t0, t0 + 30 min, t0 + 60 min, and t0 + 90 min), but each initial time t0 was shifted by 3 h (black arrows and light blue frames). The data from the Pulses study Subject 1 was used (see Materials and Methods). CORT: cortisol, k: clearance rate, kα: absorption rate. Time points of delivery (magnitude of doses): 0 min (2.3 mg/230 μL), 180 min (2.3 mg/230 μL), 360 min (0.5 mg/50 μL), 540 min (0.5 mg/50 μL), 720 min (4 mg/400 μL), 900 min (4 mg/400 μL), 1080 min (4 mg/400 μL), 1260 min (2.3 mg/230 μL). The orange dots in the diagrams represent the actual plasma cortisol measurements (which are the same in all cases), and the continuous lines (of varying colours) represent the model predictions (which slightly change depending on the set of four CORT measurements, which the calculations were based on).

**Figure A2:**
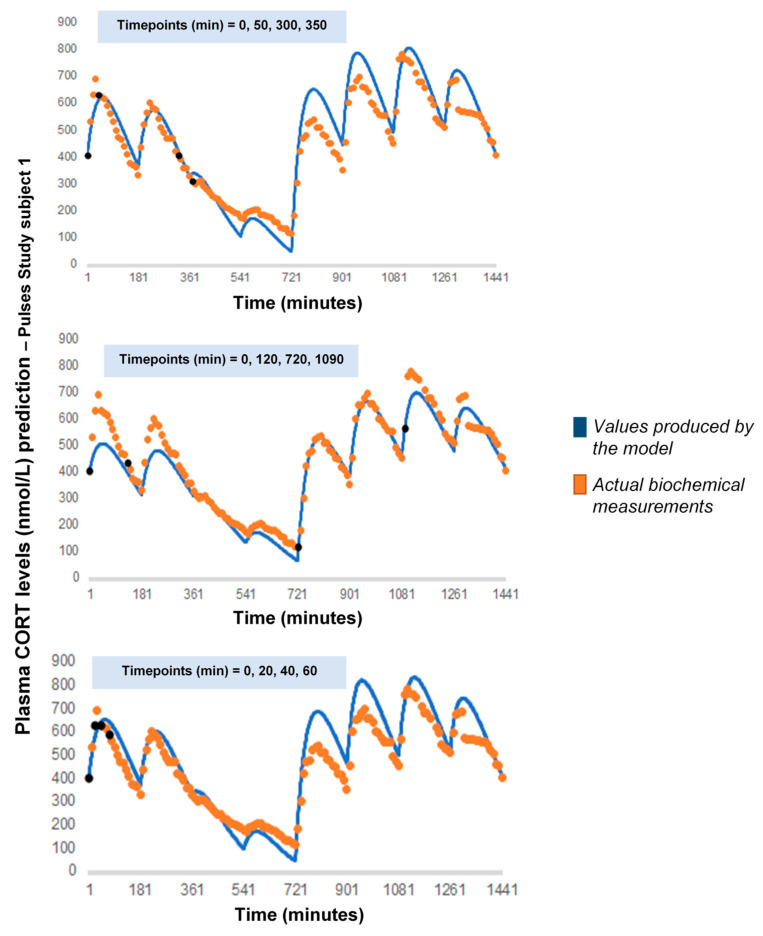
Comparison of the revised model predictions with plasma CORT data, if calculation of the initial pair of (k, kα) was based on another set of four CORT measurements. The first CORT measurement always corresponded to t0 = 0, but we changed the time distance of the remaining CORT measurements, being either short, long, or mixed (black dots in each diagram and blue frames). The data from the Pulses study Subject 1 was used (see Materials and Methods). CORT: cortisol, k: clearance rate, kα: absorption rate. Time points of delivery (magnitude of doses): 0 min (2.3 mg/230 μL), 180 min (2.3 mg/230 μL), 360 min (0.5 mg/50 μL), 540 min (0.5 mg/50 μL), 720 min (4 mg/400 μL), 900 min (4 mg/400 μL), 1080 min (4 mg/400 μL), 1260 min (2.3 mg/230 μL).

## Data Availability

No new data were created or analyzed in this study. Data sharing is not applicable to this article.

## Appendix A

Figure A1 represents a comparison of the revised model predictions with plasma CORT data, if calculation of the initial pair of (*k, k_α_*) is based on different sets of four CORT time point measurements. The time distance between the four CORT measurements was retained across the different calculations (t_0_, t_0_ + 30 min, t_0_ + 60 min, and t_0_ + 90 min), but each initial time t_0_ was shifted by 3 h (black arrows and light blue frames). The data from the Pulses study Subject 1 was used (see Materials and Methods). CORT: cortisol, k: clearance rate, kα: absorption rate. Time points of delivery (magnitude of doses): 0 min (2.3 mg/230 μL), 180 min (2.3 mg/230 μL), 360 min (0.5 mg/50 μL), 540 min (0.5 mg/50 μL), 720 min (4 mg/400 μL), 900 min (4 mg/400 μL), 1080 min (4 mg/400 μL), 1260 min (2.3 mg/230 μL). The orange dots in the diagrams represent the actual plasma cortisol measurements (which are the same in all cases), and the continuous lines (of varying colours) represent the model predictions (which slightly change depending on the set of four CORT measurements, which the calculations were based on).

## Appendix B

Figure A2 represents a comparison of the revised model predictions with plasma CORT data, if calculation of the initial pair of (*k, k_α_*) is based other sets of four CORT measurements, than those presented in Figure 4. For these calculations, the first CORT measurement always corresponded to t_0_ = 0, but we changed the time distance of the remaining CORT measurements, being either short, long, or mixed (black dots in each diagram and blue frames). The data from the Pulses study Subject 1 was used (see Materials and Methods). CORT: cortisol, k: clearance rate, *k_α_*: absorption rate. Time points of delivery (magnitude of doses): 0 min (2.3 mg/230 μL), 180 min (2.3 mg/230 μL), 360 min (0.5 mg/50 μL), 540 min (0.5 mg/50 μL), 720 min (4 mg/400 μL), 900 min (4 mg/400 μL), 1080 min (4 mg/400 μL), 1260 min (2.3 mg/230 μL).

## Appendix C

We begin from the differential equation (6) by replacing the time variable from t to s. Hence, we get:

(A1) $$\frac{dC\left(s\right)}{ds}+kC\left(s\right)=k_{a}De^{-k_{a}s}$$

We multiply this by the integrating factor $e^{ks}$ to get $\frac{d}{ds}\left(e^{ks}C\left(s\right)\right)=k_{a}De^{\left(k-k_{a}\right)s}$.

It follows, then, that we can find the function for any arbitrary time interval (0, t):

(A2) $$\int_{0}^{t}\frac{d}{ds}\left(e^{ks}C\left(s\right)\right)ds=\int_{0}^{t}k_{a}De^{\left(k-k_{a}\right)s}ds\;\Leftrightarrow$$

(A3) $$C\left(t\right)-e^{-kt}C\left(0\right)=\frac{k_{a}D}{k-k_{a}}\left(e^{-k_{a}t}-e^{-kt}\right)\;\Leftrightarrow$$

(A4) $$C\left(t\right)=D\frac{k_{a}}{k_{a}-k}\left(e^{-kt}-e^{-k_{a}t}\right)+\sigma e^{-kt}$$

where σ stands for the plasma cortisol concentration at time zero. This solution constitutes Equation (7) of the main text.

## Appendix D

We start from Equation (7):

(A5) $$C\left(t\right)=D_{1}\frac{k_{a}}{k_{a}-k}\left(e^{-kt}-e^{-k_{a}t}\right)+\sigma e^{-kt}$$

This holds for a single dosage D. Then for two dosages ($D_{1}$, $D_{2}$) we obtain the following:

(A6) $$C_{2}\left(t\right)=D_{1}\delta_{1}\left(t\right)\frac{k_{a}}{k_{a}-k}\left(e^{-k\left(t-t_{1}\right)}-e^{-k_{a}(t-t_{1}}\right)+D_{2}\delta_{2}\left(t\right)\frac{k_{a}}{k_{a}-k}\left(e^{-k\left(t-t_{2}\right)}-e^{-k_{a}\left(t-t_{2}\right)}\right)+\sigma e^{-kt}$$

where $\delta_{1}\left(t\right)$ = 0 for t < $t_{1}$ and 1 for t > $t_{1}$, $\delta_{2}\left(t\right)$ = 0 for t < $t_{2}$ and 1 for t > $t_{2}$, with $t_{1}$ = 0 and $t_{2}$ = 180 min. Extrapolating from this, we can derive a formula for the cumulative effect of an arbitrary number n of doses:

(A7) $$C_{n}\left(t\right)=\frac{k_{a}}{k_{a}-k}\left(\overset{n}{\underset{i=1}{\sum }}\delta_{i}\left(t\right)D_{i}\left(e^{-k\left(t-t_{i}\right)}-e^{-k_{a}\left(t-t_{i}\right)}\right)\right)+\sigma e^{-kt}$$

where $t_{1}$ = 0, $t_{2}$ = 180 min, $t_{3}$ = 360 min, etc.

## Appendix E

To estimate k(t), we use the following algorithm:

Set *k* = *k*_0_, *k*_0_ constant.

For (*t* = 0, *t* ≤ *t_n_*, *t*++)

(A8) $$K\left(t+1\right)=k_{0}\frac{C\left(t\right)}{C\left(0\right)}$$

if *K*(*t*+1) > *K*(*t*)

*k*(*t*+1) = *K*(*t*+1)

else *k*(*t*+1) = *K*(*t*)

if *t* = 1260

*k*(*t*) = *k*0

Return *k*(*t*)

Where *K*(*t*) is an intermediate variable used to calculate *k*(*t*). The time *t* = 1260 (12:00 p.m.) is where the reset to the initial value happens. We assume a period of 24 h, although for some profiles the resetting might take place at shorter time intervals. Our model can accommodate shorter reset periods such as 12-h long periods, although we present the 24-h period which best fits the data. It is worth noting that recent experimental data support that hepatocytes do not have a constant catabolic capacity, but rather an oscillating catabolic capacity, characterized by periods of 8, 12 or 24 h [28].

## Appendix F

Based on the assumption that a small sphere of HC solution is created within the subcutaneous tissue, it follows that the rate of absorption is dependent on the area of that sphere. Since the rate is calculated as a percentage of cortisol at a given time, we can postulate the following formula:

(A9) $$k_{a}\left(t\right)=\frac{4\pi r{\left(t\right)}^{2}}{V}\hat{v}$$

where $\hat{v}$ is assumed to be a constant.

After obtaining a time, t, the radius of the sphere is r(t):

(A10) $$k_{a}\left(t\right)=\frac{4\pi \hat{v}\left(r{\left(t\right)}^{2}\right)}{\frac{4}{3}\pi {\left(r\left(t\right)\right)}^{3}}=\frac{3\hat{v}}{r\left(t\right)}$$

Yet since r(t) is dependent on the remaining dosage $S_{c}\left(t\right)$ (taking into consideration that 1 mg of hydrocortisone is diluted in 100 μL of volume), we obtain the following:

(A11) $$100S_{c}\left(t\right)=\frac{4}{3}\pi r{\left(t\right)}^{3}\Leftrightarrow$$

(A12) $$r\left(t\right)={\left(\frac{75}{\pi }\times S_{c}\left(t\right)\right)}^{\frac{1}{3}}$$

Now, we may relate *k_α_*(*t*) to *k_α_*(0) by calculating:

(A13) $$\frac{k_{a}\left(t\right)}{k_{a}\left(0\right)}=\frac{\frac{3\hat{v}}{r\left(t\right)}}{\frac{3\hat{v}}{r\left(0\right)}}=\frac{r\left(0\right)}{r\left(t\right)}$$

It follows that $k_{a}\left(t\right)={\left(\frac{S_{c}\left(0\right)}{S_{c}\left(t\right)}\right)}^{\frac{1}{3}}\times k_{a}\left(0\right)$.

Finally, based on our initial assumptions, we can estimate the volume of the assumed sphere in the subcutaneous tissue at any time. To make calculations easier, we do that by estimating it once every minute. The use of the symbol $\hat{v}$ for the constant is deliberate. Starting from (A10) we get that:

(A14) $$k_{a}\left(t\right)=\frac{3\hat{v}}{r\left(t\right)}\;\Leftrightarrow$$

(A15) $$\hat{v}=\frac{k_{a}\left(t\right)r\left(t\right)}{3}$$

By doing a dimensional analysis of (A15) we get:

(A16) $$\hat{v}=\frac{\left[1\right]}{\left[time\right]}\times \left[length\right]=\frac{\left[length\right]}{\left[time\right]}$$

$k_{a}\left(t\right)=\frac{1}{\left[time\right]}$ is a rate and r(t) is measured in units of length. Hence, we conclude that $\hat{v}$ is a unit of velocity. To understand this, we need to note that *k_α_* represents the probability that a molecule belonging to the sphere within the subcutaneous tissue passes to the bloodstream times the number of molecules belonging to the sphere, i.e., the rate at which molecules pass from the subcutaneous tissue to the bloodstream. From a statistical mechanics point of view, the *k*_α_ rate can thus be interpreted as the group velocity $\hat{v}$ of the molecules crossing from one compartment to the other per unit length.

## Appendix G

To estimate *k_α_*(*t*) we use the following algorithm:

Set $S_{c}\left(0\right)$ = D

Set *k_α_*(0) = *k_α_*0

For (*t* = 0; *t* ≤ *t_n_*; *t* ++)

$S_{c}\left(t+1\right)$ = $S_{c}\left(t\right)$exp(−*k_α_*(*t*))

(A17) $$k_{a}\left(t+1\right)=k_{a}0{\left(\frac{S_{c}\left(t\right)}{S_{c}\left(0\right)}\right)}^{\frac{1}{3}}$$

Return *k_α_*(*t*)

Return $S_{c}\left(t\right)$
